# Phylogenetic Reassessment of Murinae Inferred from the Mitogenome of the Monotypic Genus *Dacnomys* Endemic to Southeast Asia: New Insights into Genetic Diversity Erosion

**DOI:** 10.3390/biology14080948

**Published:** 2025-07-28

**Authors:** Zhongsong Wang, Di Zhao, Wenyu Song, Wenge Dong

**Affiliations:** Yunnan Provincial Key Laboratory for Zoonosis Control and Prevention, Institute of Pathogens and Vectors, Dali University, Dali 671000, China; wangzs09@sina.com (Z.W.); zhaodi5038@163.com (D.Z.)

**Keywords:** Murinae phylogenomics, mitogenome evolution, monotypic genus, genetic diversity erosion, evolutionary constraints

## Abstract

The Millard’s rat (*Dacnomys millardi*), a threatened murid endemic to Southeast Asian montane rainforests and the sole member of its monotypic genus, faces escalating endangered risks as a Near Threatened species in China’s Biodiversity Red List. This ecologically specialized rodent exhibits diagnostic morphological adaptations—hypertrophied upper molars and cryptic pelage—that underpin niche differentiation in undisturbed tropical/subtropical forests. Despite its evolutionary distinctiveness, the conservation prioritization given to *Dacnomys* is hindered due to a deficiency of data and unresolved phylogenetic relationships. Here, we integrated morphological analyses with the first complete mitogenome (16,289 bp in size; no structural rearrangements) of *D. millardi* to validate its phylogenetic placement within the subfamily Murinae and provide novel insights into genetic diversity erosion. Bayesian and maximum likelihood phylogenies robustly supported *Dacnomys* as sister to *Leopoldamys* (PP = 1.0; BS = 100%), with an early Pliocene divergence (~4.8 Mya, 95% HPD: 3.65–5.47 Mya). Additionally, based on its basal phylogenetic position within Murinae, we propose reclassifying *Micromys* from Rattini to the tribe Micromyini. Codon usage bias analyses revealed pervasive purifying selection (Ka/Ks < 1), constraining mitogenome evolution. Genetic diversity analyses showed low genetic variation (*CYTB*: *π* = 0.0135 ± 0.0023; *COX1*: *π* = 0.0101 ± 0.0025) in fragmented populations. We propose three new insights into this genetic diversity erosion. (1) Evolutionary constraints: genome-wide evolutionary conservation and shallow evolutionary history (~4.8 Mya) limited mutation accumulation. (2) Anthropogenic pressures: deforestation-driven fragmentation of habitats (>20,000 km^2^/year loss since 2000) has reduced effective population size, exacerbating genetic drift. (3) Ecological specialization: long-term adaptation to stable niches favored genomic optimization over adaptive flexibility. These findings necessitate suitable conservation action by enforcing protection of core habitats to prevent deforestation-driven population collapses and advocating IUCN reclassification of *D. millardi* from Data Deficient to Near Threatened.

## Simple Summary

Millard’s rat, a rare mountain forest rodent in Southeast Asia, faces endangerment due to unclear evolutionary features and genetic diversity loss. Using morphological traits and complete mitochondrial DNA decoding for the first time, we discovered the following: (1) its closest relative is *Leopoldamys* rats, splitting 4.8 million years ago; (2) populations show critically low genetic variation, driven by three influencing factors: evolutionary constraints limiting mutations, forest loss fragmenting habitats (>20,000 km^2^/year since 2000), and ecological specialization reducing adaptability; (3) this requires reclassifying *Micromys* rats in the Murinae phylogenetic relationships. Urgent conservation actions are proposed: protect core forest habitats to halt genetic decline and upgrade the species to “Near Threatened” on the IUCN Red List.

## 1. Introduction

The Millard’s rat (*Dacnomys millardi* Thomas, 1916), the sole extant species of the monotypic genus *Dacnomys* (Rodentia: Muridae), represents an evolutionarily distinct lineage within the Southeast Asia biodiversity hotspot. Its disjunct distribution spans from eastern Nepal and northeastern India (type locality: Darjeeling, West Bengal) to northern Laos and northwestern Vietnam [1], with isolated populations recently documented in southern Vietnam [2] and China’s Yunnan-Tibet border region [3]. This fragmented distribution range and rarity of *D. millardi* suggest it has specific ecological requirements that are met only in pristine habitats [1,4]. Its diet, consisting of plant seeds, roots, stems, and buds [4], indicates a role in seed dispersal and potentially in shaping plant community structure [5]. The species is restricted to undisturbed tropical/subtropical montane rainforests at 500–3500 m elevation [1,4]. This limited distribution range coincides with reduced effective population sizes and accelerating habitats loss—synergistic drivers of its Near Threatened status in China’s Biodiversity Red List [6], despite its IUCN Data Deficient classification [7]. The absence of this species in disturbed areas [1], combined with the threat of deforestation [8], underscores its dependency on a healthy ecosystem. Thus, this species is not only an indicator of ecosystem health but also plays a part in sustaining the ecological processes vital for a balanced environment. However, Southeast Asia’s tropical forests exhibit the world’s highest deforestation rates (annual loss exceeding 20,000 km^2^ since 2000) [8,9], a critical threat compounded by *D. millardi*’s absence in disturbed habitats [1], reflecting low ecological plasticity and heightened population vulnerability.

Persistent controversies in the subfamily Murinae systematics, particularly regarding the phylogenetic position of *Micromys* (Dehne, 1841), stem from two critical limitations: (1) the absence of genomic data for key lineages, hindering precise estimation of phylogeny and divergence times; (2) overreliance on short gene fragments [e.g., cytochrome b (*CYTB*), retinol binding protein 3 (*RBP3*), growth hormone receptor (*GHR*)] [10] or morphological traits in taxonomic studies [11]. In contrast, complete mitogenomes provide unequivocal evidence of a conserved order of homologous genes over hundreds of millions of years and enhanced resolution to resolve evolutionary relationships among closely related species [12,13]. Those with elevated substitution rates (~4.8 × 10^−8^ substitutions/site/year) [14], and reduced recombination—offer superior phylogenetic sensitivity for rodent clades [15,16]. Hence, the past decade has witnessed a paradigm shift in mammalian phylogenetics, with researchers increasingly prioritizing complete mitogenome sequencing over analyses of single nuclear loci or partial mitochondrial DNA fragments [17,18,19,20].

Notably, mitogenomic datasets have played a pivotal role in clarifying contentious branching patterns among placental mammal orders, contributing critical molecular evidence that solidified several previously debated clades [21,22]. Rodent recent studies exemplified this progression: İbiş et al. (2020) [23] addressed the unresolved phylogenetic position of *Prometheomys schaposchnikowi* (Satunin, 1901) by sequencing its complete mitogenome and integrating 58 Arvicolinae mitogenomic sequences. Yu et al. (2022) [24] reconstructed the Holocene dispersal dynamics of the invasive black rat (*Rattus rattus* Linnaeus, 1758) via ancient genomic approaches, combining a de novo reference genome with 103 mitogenomes and 39 nuclear genomes from 1st–17th centuries CE specimens across Europe and North Africa. These attributes position mitogenomes as robust phylogenetic markers for resolving relationships across taxonomic ranks. Additionally, Liu et al. (2025) [25] reconstructed phylogeny and evolutionary history of Murinae through integration of four newly sequenced *Vernaya* (Anthony, 1941) mitogenomes alongside 36 published Murinae mitogenomes and 17 *CYTB* sequences, thereby validating the new tribe Vernayaini (Liu, Zhao, Liu & Chen, tribe nov.). However, although the complete mitogenomes provided enhanced phylogenetic resolution through their comprehensive nucleotide data, enabling robust inferences of evolutionary relationships at both shallow and deep taxonomic scales [26,27,28], we note the limitations of mitogenomic analyses, particularly methodological constraints including in-complete lineage sorting, introgression artifacts, and hybridization signals [26,29], and its restricted representation of genome-wide evolutionary processes.

While early studies proposed the delineation of distinct subspecies (*D. m. wroughtoni* Thomas, 1922; *D. m. ingens* Osgood, 1932), which is inconsistent with the current taxonomic system of *D. millardi* as a monotypic species [1,3,4,6,30]. Research on the poorly studied genus *Dacnomys* has been severely limited due to its narrow distribution, small effective population size, and apparent absence from disturbed rainforests [1,6]. Prior to 2012, the phylogenetic position of *Dacnomys* remained unresolved, the earliest studies on the phylogenetic framework for *Dacnomys* primarily focused on limited genetic markers: Mitochondrial genes (*CYTB*, *COX1*) were investigated by Balakirev et al. (2012) [31]. Steppan et al. (2017) [10] first established its placement within the Rattini tribe using single genes (*CYTB*, *RBP3*, *GHR*), demonstrating a close evolutionary relationship between *Dacnomys* and *Niviventer* (Marshall, 1976). Subsequently, Abramov et al. (2017) [2] expanded the known distribution of the genus through new records in southern Vietnam and conducted a comprehensive morphological and genetic analysis of population-level variation using extensive museum specimens. Notably, no study has yet comprehensively employed complete mitogenome data to elucidate the phylogenetic relationships and refine divergence time estimation of *D. millardi*. This gap considerably impeded a comprehensive understanding of its taxonomic variation, evolutionary rate dynamics, and biodiversity patterns. Additionally, limited cranial differentiation and low genetic diversity (*CYTB* difference not exceeding 0.023) across populations suggested that synergistic effects of evolutionary constraints and anthropogenic pressures underpin its genetic homogeneity [2]. However, the factors driving this homogeneity warrant further elucidation. This study integrated morphological characterization with the first complete mitogenome of *Dacnomys* to validate its phylogenetic placement and reassess Murinae taxonomy. By analyzing mitogenome structural conservation, habitats fragmentation, and evolutionary histories, we explored potential influencing factors contributing to its genetic diversity erosion. Our findings underscored the urgent need for habitats conservation and genetic management of this threatened lineage while advancing understanding of evolutionary constraints shaping biodiversity in anthropogenically fragmented landscapes.

## 2. Materials and Methods

### 2.1. Specimen Collection and Identification, DNA Extraction and Sequencing

**Figure 1:**
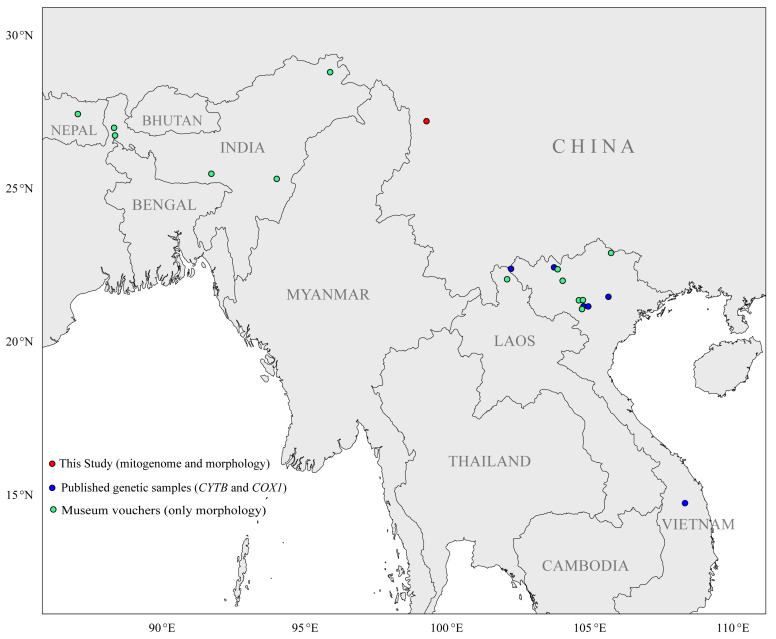
Biogeographic distribution of *Dacnomys millardi* occurrence records classified by evidence type. Here, Red points = whole-mitogenome sequenced specimens with morphological validation (this study), Blue points = published genetic samples (partial *CYTB*/*COX1* sequences), Green points = museum vouchers with only morphology. Data are from new occurrence records of southwestern China (this study), genetic samples and museum voucher records of Abramov et al. (2017) [2].

Two adult *D. millardi* individuals were collected from montane rainforests in Weixi Lisu Autonomous County, Yunnan Province, China (27.18° N, 99.28° E; 3000 m a.s.l.). Field coordinates, elevation, and vegetation type were georeferenced for each specimen. Standard morphometric parameters—body mass (g), head-body length (mm), tail length (mm), hindfoot length (mm), and ear length (mm)—were recorded in situ. The species distribution map integrated georeferenced occurrence records from Abramov et al. (2017) [2] with documented distribution ranges in Wei et al. (2022) [3] and Wilson et al. (2017) [1] (Location information: Table S1, Figure 1). Spatial visualization was performed in R v4.3.1 using the *rnaturalearth* v1.0.1 package with the World Geodetic System 1984 (WGS84) coordinate reference system. Specimens with intact craniodental features and undamaged pelage were preserved as vouchers following the Animal Ethics Committee guidelines. The specimens (voucher IDs: E420001, E420002) were deposited in the Institute of Pathogens and Vectors, Dali University (Dali, China). Tissue samples (muscle, liver, spleen) were excised under sterile conditions, preserved in 95% ethanol, and stored at –80 °C. Specimen identification was confirmed by cranial morphology and *CYTB* barcoding (primers L14725_hsw1: 5′-ATGACATGAAAAATCATCGTTGT-3′; H15915_hsw1: 5′-TCYCC ATTTCTGGTTTACAAGACC-3′) [32], with taxonomic references to Wei et al. (2022) [3] and Wilson et al. (2017) [1].

Total genomic DNA was extracted from ethanol-preserved muscle tissue using the DNeasy Blood and Tissue Kit (QIAGEN, Redwood City, CA, USA). Libraries with 300–500 bp inserts were prepared using the Illumina TruSeq Nano DNA Kit (Illumina, San Diego, CA, USA) and sequenced on an Illumina NovaSeq 6000 platform (2 × 150 bp PE; >100× coverage) [33] by Shanghai Winnerbio Technology Co., Ltd. (Shanghai, China).

### 2.2. Sequence Assembly and Annotation

Raw sequencing reads were quality-filtered using FastQC v0.20.1 [34] with Phred scores ≥ 36. De novo assembly of the *D. millardi* mitogenome was performed by a dual-pipeline approach: (1) MITOZ v2.3 [35] for seed-based iterative extension, and (2) Geneious Prime v11.1.5 [36] for reference-guided alignment (NCBI Murinae mitogenomes as templates). Assembly confidence was validated via BWA v0.7.17 [37] read mapping and SAMtools v0.1.20 [38] depth analysis, achieving ≥ 100× coverage to ensure single-base accuracy (error rate < 0.01%).

Gene annotation combined automated and manual curation. (1) Structural annotation: protein-coding genes (PCGs), ribosomal RNAs (rRNAs), transfer RNAs (tRNAs), and control regions (D-loop) were identified using MITOS2 with the vertebrate genetic code [39]. (2) tRNAs validation: tRNAs genes were predicted using tRNAscan-SE v2.0 [40], ARWEN v1.2 [41], and manual covariance models. (3) Manual refinement: annotations were cross-validated against NCBI Murinae homologs (BLASTn E-value < 1 × 10^−10^) and adjusted for conserved start/stop codons. The circular mitogenome map was visualized using OGDRAW v1.3.1 [42] with default parameters.

### 2.3. Sequence Analyses, Genetic Diversity and Morphological Visualization 

The molecular analyses integrated novel and published data. We newly sequenced a complete mitogenome from one *D. millardi* specimen (voucher ID: E420001; GenBank accession: PQ359525), which was combined with 18 published mitochondrial DNA sequences from Abramov et al. (2017) [2] [nine *CYTB* and nine cytochrome oxidase subunit 1 (*COX1*); accessions: Table S2] to re-analyze intraspecific genetic diversity across the distribution range of *Dacnomys*.

Genetic divergence among populations from southwestern China (this study), northern and southern Vietnam was quantified using Tamura-3-parameter pairwise distances for the *CYTB* gene [43], selected for its evolutionary clock consistency in Murinae [44]. Intraspecific genetic diversity was quantified through haplotype diversity (*Hd*), nucleotide diversity (*π*), and Tajima’s *D* statistics in DnaSP v6.12.03 [45] for the mitochondrial genes *CYTB* and *COX1*. Statistical significance for Tajima’s *D* was defined as |*D*| > 2.0 [46], with neutrality tests conducted under an infinite-sites model [47].

Nucleotide composition and codon usage bias of the *D. millardi* mitogenome were analyzed in MEGA v11.0.13 [48] with default parameters. Selection pressure analysis employed DnaSP v6.12.03 [45] to calculate non-synonymous/synonymous substitution ratios (*ω* = Ka/Ks) of 13 PCGs in the *D. millardi* mitogenome. Strand asymmetry was reflected by AT- and GC-skew values: AT-skew = (A − T)/(A + T) and GC-skew = (G − C)/(G + C) [49]. 

Codon usage patterns of the *D. millardi* mitogenome were characterized by: (1) Relative synonymous codon usage (RSCU) [50], effective number of codons (ENC)-plot analysis [51] and correspondence analysis (COA) [52] of codon frequencies in CodonW [53] (excluding termination codons); (2) neutrality curve (GC12 vs. GC3s) [54] using Python v3.10.12 (with SciPy v1.11.1); (3) parity rule 2 (PR2) [55] plot of purine/pyrimidine bias with R v4.3.1 (via Biostrings 2.68.1 software package).

Morphological visualization of the specimen (voucher IDs: E420001) involved focus-stacked cranial and pelage images captured with a Canon EOS R6 camera system, post-processed in Adobe Photoshop 2023 (v24.7.0) for brightness/contrast normalization (≤5% adjustment) without structural modifications.

### 2.4. Phylogenetic Reconstruction

A dataset of 68 species and subspecies (16 genera, eight tribes, two subfamilies) in the family Muridae was created from GenBank (Species information: Table S3), supplemented with the newly sequenced *D. millardi* mitogenome. Two sciurid species (*Ratufa bicolor* Sparrman, 1778; *Pteromys volans* Linnaeus, 1758) served as outgroups. 13 PCGs and two rRNAs were extracted and concatenated using PhyloSuite v1.2.3 [56] to generate a protein-coding and ribosomal RNA gene sequence (PCGRNA) matrix. Gene sequences were aligned with MAFFT v7.313 [57] under the G-INS-i algorithm, followed by manual refinement in BioEdit v7.2.5 (https://bioedit.software.informer.com/ (accessed on 5 June 2025)). Ambiguous regions were pruned using Gblocks v0.91 [58] with relaxed parameters. The presence of phylogenetic signal was evaluated with a substitution saturation analysis using the Xia test [59] in the DAMBE v7.3.32 [60] for the whole alignment of the PCGRNA. The test confirmed the absence of significant substitution saturation in PCGRNA, supporting dataset’s reliability for phylogenetic inference (Table S4).

To ensure methodological rigor and topological robustness, we employed two complementary approaches: (1) Bayesian Inference (BI): The concatenated matrix was analyzed in MrBayes v3.2.6 [61] under the best-fit GTR + F + I + G4 model (partitioned by codon in PartitionFinder v2.1.1) [62]. Four independent MCMC runs (1 × 107 generations, sampling every 1000) achieved convergence (average standard deviation of split frequencies < 0.01). The first 25% trees were discarded as burn-in, and a 50% majority consensus tree was generated. (2) Maximum Likelihood (ML): IQ-TREE v2.2.0 [63] implemented the GTR + F + R6 model (partitioned by codon in ModelFinder [64]) with 1000 ultrafast bootstrap replicates. Branch supports were validated via SH-aLRT (threshold: 80%) [65] and UFboot (threshold: 95%) [66]. Final trees were annotated in iTOL v6.8.1 [67] and formatted for publication using Adobe Illustrator 2023 (v27.9.1) without altering topological relationships.

### 2.5. Divergence Time Estimation

Divergence times were estimated using Bayesian molecular dating in BEAST v2.6.7 [68] under an uncorrelated lognormal relaxed clock model with a Yule speciation prior. Four fossil-calibrated nodes (Table S5) were constrained using lognormal prior following Murinae fossil records from Aghová et al., (2018) [69]. Two independent MCMC runs of 500 million generations each were conducted, sampling parameters and trees every 10,000 generations. Convergence was confirmed in Tracer v1.7.2 [70] through effective sample sizes (ESS > 200 for all parameters) and potential scale reduction factors (PSRF ≈ 1.0) [71]. Post-burnin trees (25% discarded) were combined in LogCombiner v2.6.7 [68] and a maximum clade credibility tree was estimated in TreeAnnotator v2.6.7 [72], retaining nodes with Bayesian posterior probabilities (PP) ≥ 0.95 and maximum likelihood bootstrap supports (BS) ≥ 70% from prior analyses [73,74]. Final chronograms were visualized in TVBOT v2.6.1 [75] with geological timescales standardized from International Commission on Stratigraphy (http://www.stratigraphy.org/ICSchart/ChronostratChart2024-12.pdf (accessed on 5 June 2025)).

## 3. Results

### 3.1. Morphological Characteristics of D. millardi

**Figure 2:**
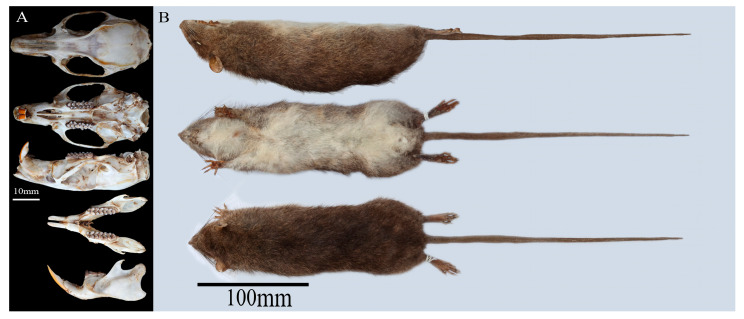
Morphological characterization of *Dacnomys millardi* (specimen E420001). (**A**) The cranium and mandible in dorsal, ventral, and lateral views; (**B**) the study skin in dorsal, ventral, and lateral views.

The adult female specimen (voucher ID: E420001; body mass: 475.00 g; head-body length: 270.00 mm; tail length: 305.00 mm; hindfoot length: 50.00 mm; ear length: 24.00 mm) exhibited diagnostic morphological traits distinguishing it from sympatric *Leopoldamys* species (Figure 2 and Table S6). External morphology was characterized by the following: (1) a proportionally elongated tail (112% of head-body length), uniformly pigmented without a terminal tuft; (2) dorsal pelage transitioning from short, grizzled hairs (grayish-brown variegated with subtle light-yellow spots) to ventrolateral white guard hairs; (3) ventral fur bicolored (grayish-brown basal third, dark cream distal two-thirds); (4) cream-colored throat, axillary, and inguinal regions; (5) white plantar surfaces with light-brown dorsal limb pigmentation; and (6) four pairs of mammae arranged in pectoral and inguinal pairs.

Craniodental features included the following: (1) a robust skull (greatest length: 51.63 mm; upper molar row: 11.29 mm) with flattened auditory bullae (10% of cranial length); (2) incisive foramina terminating anterior to the first upper molar; (3) palatal shelf extending posteriorly between the second and third upper molars; and (4) a molar formula of 1.0.0.3/1.0.0.3 = 16, consistent with Myomorpha dental archetype.

### 3.2. Mitogenome Architecture of D. millardi

**Figure 3:**
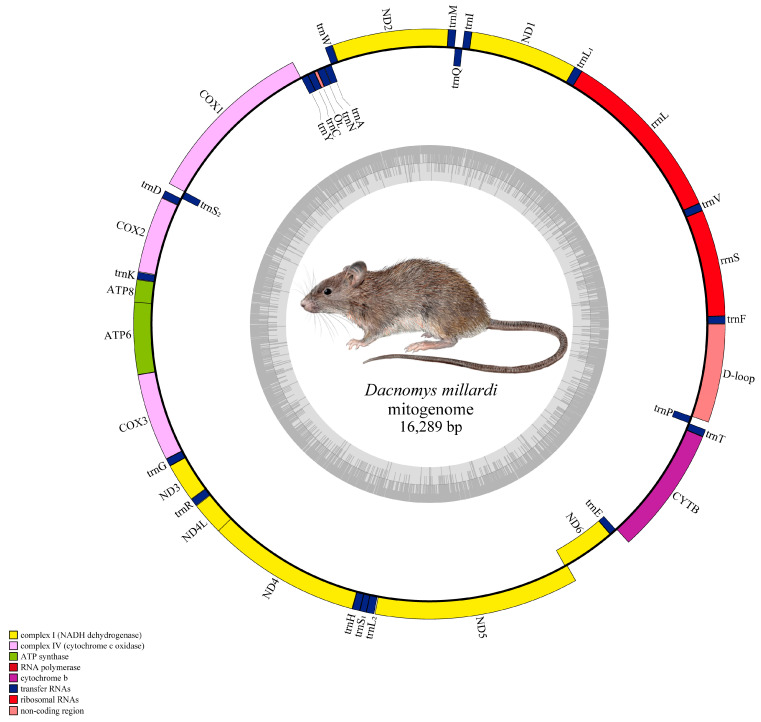
Circular mitogenome map of the *Dacnomys millardi*. The morphological illustration of *D. millardi* (inset) was adapted from Wilson et al. (2017, Figure 639) [1].

The complete mitogenome of *D. millardi* (16,289 bp) exhibits typical metazoan organization, comprising 37 genes (13 PCGs, 22 tRNAs, two rRNAs), one non-coding region (D-loop), and a light-strand replication origin (OL) (Figure 3). Genes strand distribution followed the ancestral murid pattern: nine genes (*ND6* and eight tRNAs: *trnQ*, *trnA*, *trnN*, *trnC*, *trnY*, *trnS*_2_, *trnE*, *trnP*) were encoded on the light strand, with the remainder on the heavy strand. The *D. millardi* mitogenome had 10 overlapping regions (1–43 bp), with the longest overlap (43 bp) between *ATP8* and *ATP6*; and 13 intergenic spacers (1–6 bp), the largest (6 bp) located between *trnL*_2_ and *ND5* (Table 1). Nucleotide composition was AT-biased (62.0% AT; 38.0% GC), with skew values of AT = 0.094 and GC = −0.363 (Table 2). The D-loop (881 bp) spanned *trnP* to *trnF*, containing conserved motifs associated with replication initiation. The OL (34 bp) was embedded within the WANCY tRNA cluster (*trnW-trnY*), forming a stem-loop structure critical for light-strand replication. The 22 tRNAs were interspersed between PCGs and rRNAs, with *rrnS* and *rrnL* flanked by *trnF* and *trnL*_1_, separated by *trnV*.

### 3.3. tRNAs and rRNAs Structural Features

**Figure 4:**
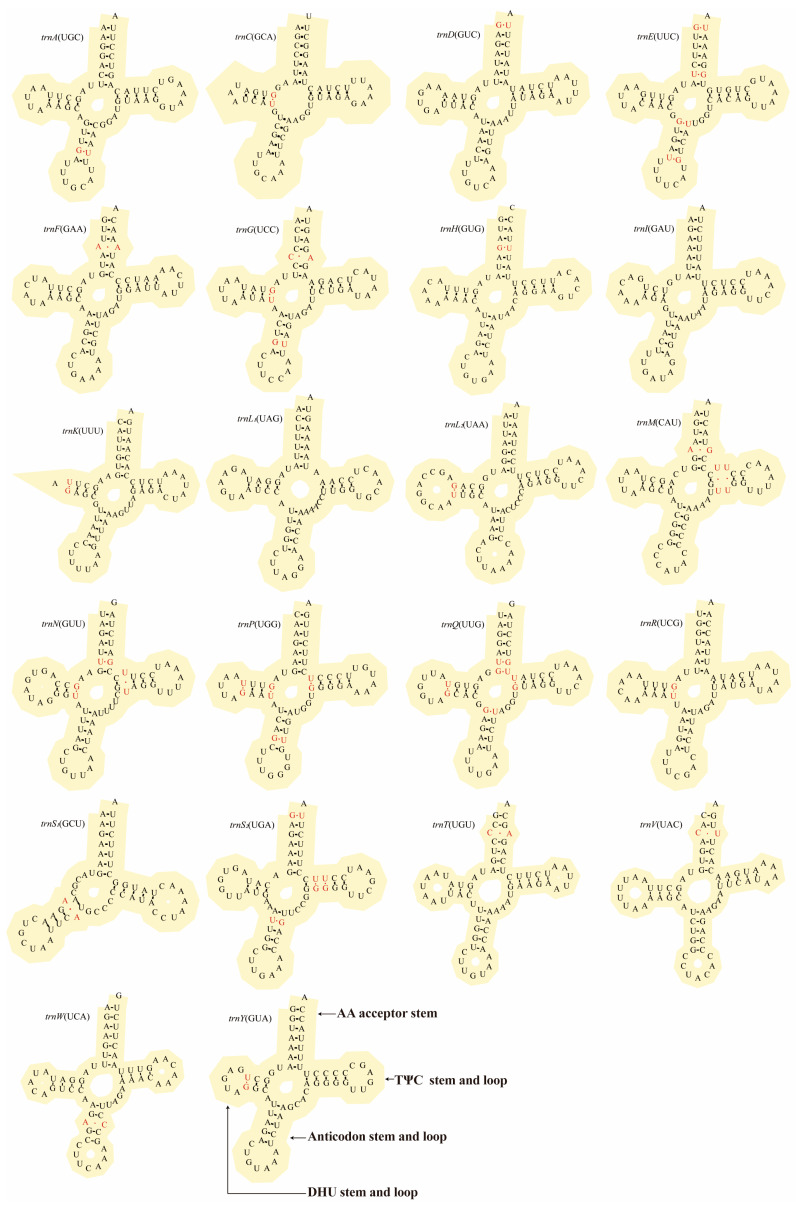
Predicted cloverleaf secondary structures of 22 tRNAs in *Dacnomys millardi* mitogenome (GenBank: PQ359525). The structures of tRNAs were identified using tRNAscan-SE v2.0 and ARWEN v1.2. Anticodons were indicated by bracket annotations. Watson–Crick pairings are denoted by dashes (−); non-Watson–Crick pairings marked with red dots (·).

The mitogenome of *D. millardi* encoded 22 tRNAs (eight light-strand, 14 heavy-strand) and two rRNAs (*rrnS*: 954 bp; *rrnL*: 1576 bp), with *rrnS* and *rrnL* positioned between *trnF* and *trnL*_1_ and separated by *trnV* (Table 1). The tRNA lengths ranged from 59 bp (*trnS*_1_) to 75 bp (*trnL*_1_) (mean ± SD: 68.32 ± 2.98 bp; Table 1), and all tRNAs were predicted to form canonical cloverleaf structures (Figure 4), except for *trnS*_1_ (lacking the dihydrouridine [DHU] arm) and *trnK* (retaining a 3 bp DHU stem but lacking the DHU loop). The tRNA anticodon usage pattern of *D. millardi* was identical to the ancestral mammalian lineage. Secondary structure analysis revealed 39 non-Watson–Crick pairings across 20 tRNAs (Figure 4), predominantly involving G-U wobble pairs (29 instances in 14 tRNAs) and mismatches (A-C, U-U, A-A, A-G, C-U; 10 total). Anomalous pairs were detected in amino acid acceptor arms (11 mismatches), DHU arms (nine), TΨC arms (five), and anticodon arms (eight), while *trnI* and *trnL*_1_ displayed perfect Watson–Crick pairings. Conserved DHU stems (2–4 bp) and TΨC stems (5 bp, except for *trnW* and *trnL*_1_ with 4 bp) reflected structural constraints critical for translational fidelity (Figure 4).

### 3.4. Protein-Coding Genes, Codon Usage, and Genetic Diversity

**Figure 5:**
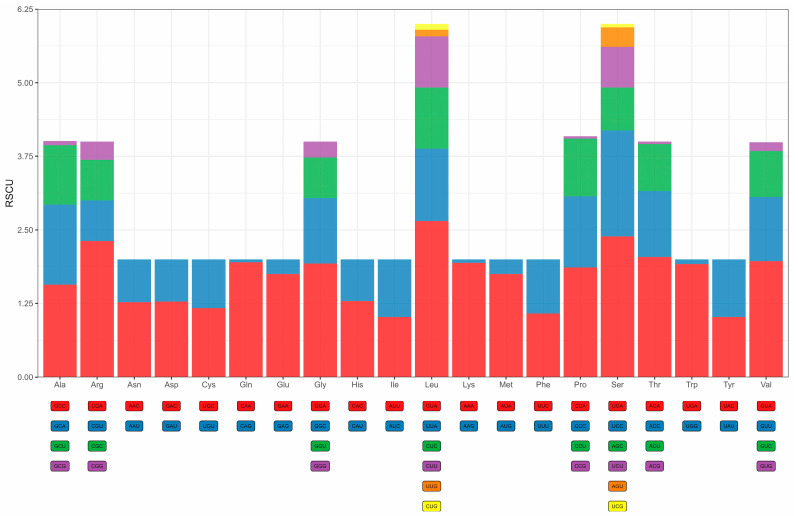
The relative synonymous codon usage (RSCU) patterns for 13 protein-coding genes (PCGs) in the *Dacnomys millardi* mitogenome.

**Figure 6:**
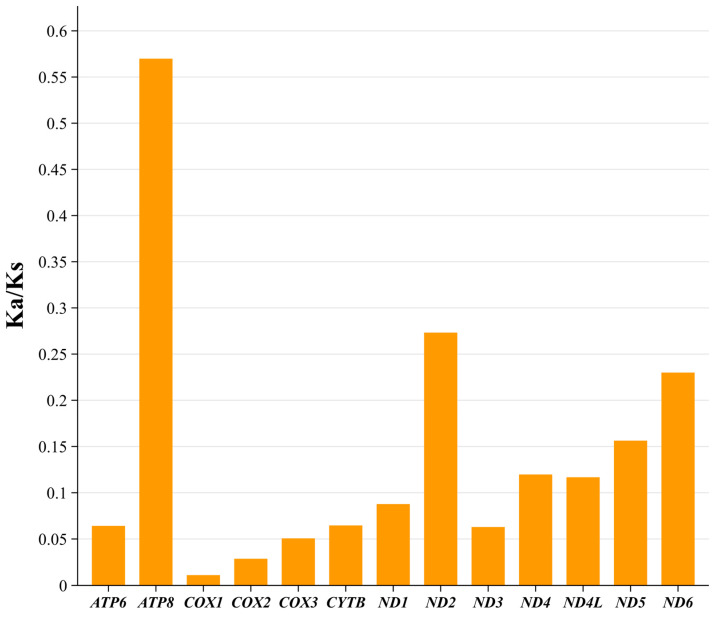
Selective pressure analysis of 13 protein-coding genes (PCGs) in the *Dacnomys millardi* mitogenome. Ka/Ks ratios were calculated for 13 PCGs using the complete mitogenome (GenBank: PQ359525) with *Ratufa bicolor* (NC_023780) and *Pteromys volans* (NC_019612) as outgroups in DnaSP v6.12.03 (default parameters).

The length of 13 PCGs in the *D. millardi* mitogenome was 11,400 bp, accounting for 69.9% of the total mitogenome length (16,289 bp). Nucleotide composition of PCGs was A = 31.6%, T = 29.7%, C = 27.0%, and G = 11.7%, yielding moderate AT bias (AT content = 61.3%; AT-skew = 0.031; GC-skew = −0.396; Table 2). All PCGs initiated with standard ATN start codons, while termination signals exhibited four patterns: canonical TAA (eight genes), incomplete T-- (*CYTB*, *COX3*, *ND4*), TAG (*ND1*), and AGA (*ND2*) (Table 1). RSCU analysis of 3792 codons revealed strong bias (Figure 5; Table 3), with 29 preferred codons (RSCU > 1) representing 74.2% of total usage (2815/3792). Highly overrepresented codons (RSCU > 1.6; 13 types, 1493 occurrences) included CUA (for Leucine, Leu), CGA (for Arginine, Arg), ACA (for Threonine, Thr), and UCA (for Serine, Ser), while 15 underrepresented codons (RSCU < 0.6; 124 occurrences) reflected translational selection against energetically costly amino acids. Purifying selection dominated mitogenome evolution, as evidenced by Ka/Ks ratios < 1 for all PCGs (Figure 6), ranging from *COX1* (Ka/Ks = 0.03) to *ATP8* (Ka/Ks = 0.57).

**Figure 7:**
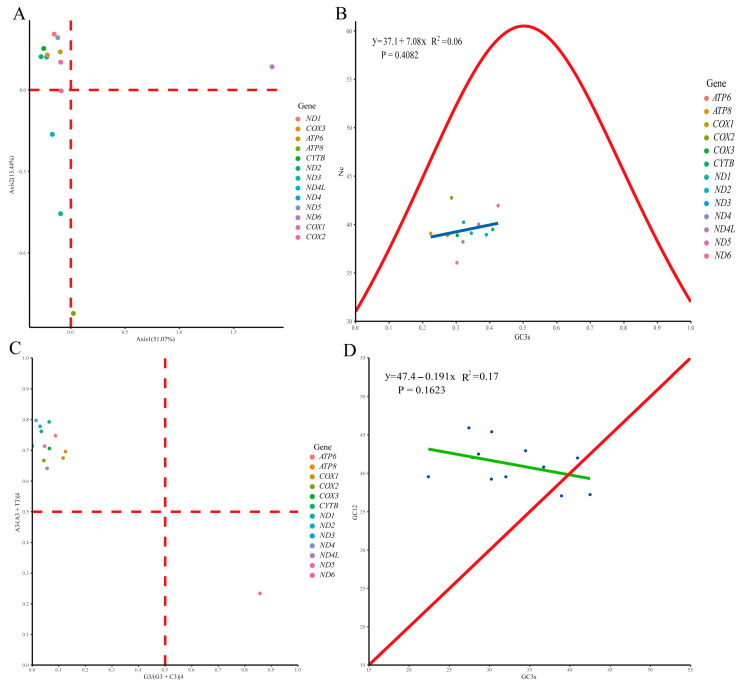
Multivariate analysis of codon usage bias of 13 protein-coding genes (PCGs) in the *Dacnomys millardi* mitogenome. (**A**) Correspondence analysis (COA) of codon frequencies (Axis 1: the first principal component; Axis 2: the second principal component); (**B**) effective number of codons (ENC)-plot (GC3s: GC content of the third position of synonymous codon; Nc: effective number of codons; the linear expression y = 37.1 + 7.08x is shown as a blue line in the figure, *R*^2^ represents the correlation, and *p* represents the significant difference); (**C**) parity rule 2 (PR2) plot of purine/pyrimidine bias; and (**D**) neutrality curve (GC12: average value of the first and second positions of synonymous codon; the linear expression y = 47.4 − 0.191x is shown as a green line in the figure, blue dots represent protein-coding genes).

Codon usage bias in the *D. millardi* mitogenome was analyzed through COA, ENC-plot, PR2, and neutrality curve (Figure 7). COA revealed heterogeneous codon usage patterns across PCGs, with most genes dispersed across the ordination space, except for *COX3* and *ND4*, which clustered closely together (Figure 7A). ENC values ranged from 36.05 (*ND6*) to 42.76 (*COX2*), with all points below the expected standard curve (Figure 7B), indicating predominant natural selection over mutation–drift equilibrium. PR2 analysis showed significant deviation from the parity rule central point (0.5, 0.5), with 12 PCGs exhibiting A/C bias and *ND6* showing G/T bias (Figure 7C), consistent with COA and ENC results. Neutrality curve regression (GC12 vs. GC3s) yielded a non-significant slope (|*r*| < 0.2, *p* > 0.05; Figure 7D), further supporting natural selection as the primary driver of codon usage variation.

Genetic diversity analysis among *D. millardi* populations from southwestern China (this study), northern and southern Vietnam displayed low genetic difference (*CYTB* divergence ≤ 0.021; Table S7). Additionally, *D. millardi* exhibited high haplotype diversity coupled with low nucleotide diversity (*CYTB*: *Hd* = 0.978 ± 0.054, *π* = 0.0135 ± 0.0023; *COX1*: *Hd* = 0.844 ± 0.103, *π* = 0.0101 ± 0.0025); neutrality tests yielded negative values (*CYTB*: Tajima’s *D* = −0.40094; *COX1*: Tajima’s *D* = −0.48964), although these deviations were not statistically significant (*p* > 0.05; Table S8).

### 3.5. Phylogenetic Analysis and Divergence Time Estimation

**Figure 8:**
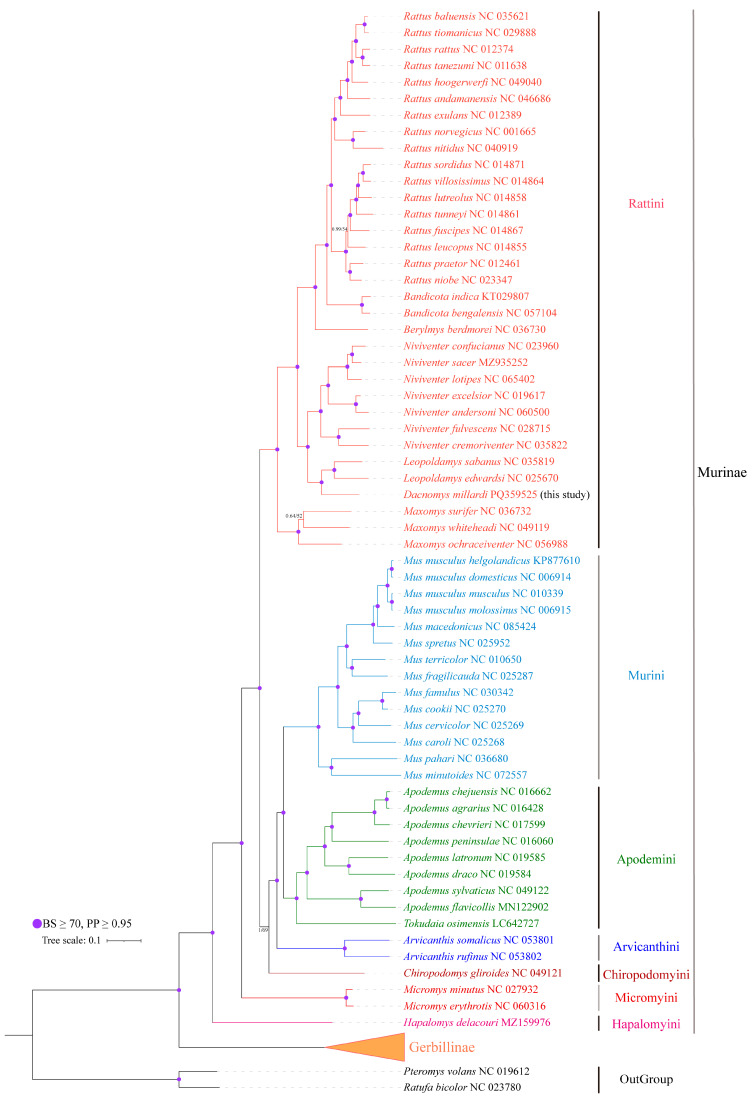
Phylogenetic reconstruction of Murinae based on partitioned mitochondrial PCGRNA alignment (13,735 bp). Purple points denote nodes with strong concordant support (BI, PP ≥ 0.95 and ML, BS ≥ 70%); the remaining nodes show BI posterior probabilities (left) and ML bootstrap supports percentages (right). The seven tribes of Murinae are marked by different colors. *Ratufa bicolor* and *Pteromys volans* were used as the outgroups.

Phylogenetic position of *D. millardi* within Muridae yielded congruent topologies with strong nodal support (PP = 1.00; BS = 100%; Figure 8). The phylogenetic relationship among different genera of Murinae were as follows: ((((((*Rattus* + *Bandicota*) +*Berylmys*) + (*Niviventer* + (*Leopoldamys* + *Dacnomys*))) + *Maxomys*) + (((*Mus* + (*Apodemus* + *Tokadaia*)) + *Arvicanthis*) + *Chiropodomys*)) + *Micromys*) + *Hapalomys*. Murinae formed a monophyletic clade (PP = 1.00; BS = 100%), with *Micromys* positioned as the basal lineage (PP = 1.00; BS = 100%) within Murinae. *D. millardi* exhibited a sister relationship with *Leopoldamys* (PP = 1.00; BS = 100%), collectively forming a clade with *Niviventer*. The *Apodemus–Tokudaia–Mus* alliance formed a monophyletic group, with *Tokudaia* sister to *Apodemus* and *Mus* as their distant relative. *Hapalomys* occupied a basal Murinae position (PP = 1.00; BS = 91%), although with lower ML support compared to *Micromys*.

**Figure 9:**
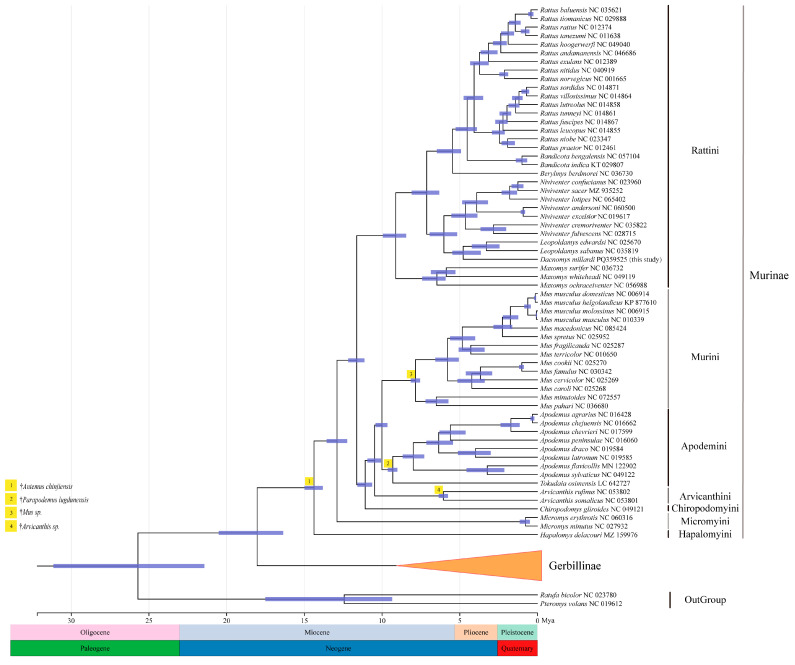
Time-calibrated phylogeny of the subfamily Murinae (Rodentia: Muridae) inferred from mitogenomes using BEAST v2.6.7. Nodes show medians of times to MRCA; node bars indicate 95% HPD intervals. Arabic numerals in yellow squares indicate positions of four fossil constraints selected by multiple-step evaluation and used for final analysis (see Table S5 for more details).

A molecular dating analysis using four fossil calibrations revealed that the most recent common ancestor (MRCA) of Murinae emerged in the Middle Miocene (~14.3 million years ago [Mya], 95% highest posterior density [HPD]: 13.81–14.98 Mya), with the Murinae–Gerbillinae split occurring earlier in the Early Miocene (~18.0 Mya, 95% HPD: 16.37–20.51 Mya). Within Murinae, three primary lineages diverged in the Middle Miocene: Hapalomyini, Micromyini (~12.9 Mya, 95% HPD: 12.24–13.57 Mya), and core Murinae. Subsequent radiations included the Chiropodomyini–Murini–Apodemini–Arvicanthini split at ~11.0 Mya (95% HPD: 10.64–11.59 Mya), followed by tribe-level divergences within Apodemini (~9.3 Mya, 95% HPD: 7.90–11.27 Mya), Murini (~7.8 Mya, 95% HPD: 7.95–9.84 Mya), and Arvicanthini (~6.0 Mya, 95% HPD: 4.59–6.77 Mya). The Rattini tribe diverged from other Murinae ~9.1 Mya (95% HPD: 8.45–9.94 Mya). The *Dacnomys–Leopoldamys* divergence occurred in the Early Pliocene (~4.8 Mya, 95% HPD: 3.65–5.47 Mya), followed by *Niviventer* (~4.6 Mya, 95% HPD: 3.86–5.54 Mya) and *Berylmys* (~5.5 Mya, 95% HPD: 4.92–6.48 Mya). The *Rattus–Bandicota* split was resolved at ~4.0 Mya (95% HPD: 3.89–5.27 Mya), with intrageneric diversification in *Rattus* continuing into the Pleistocene (~0.4 Mya, 95% HPD: 0.27–0.60 Mya). These results aligned with Miocene–Pliocene climatic shifts driving murid adaptive radiation (Figure 9).

## 4. Discussion

### 4.1. Evolutionary Conservation and Structural Features in the D. millardi Mitogenome

We presented the first complete mitogenome assembly for the monotypic genus *Dacnomys*, filling a critical gap in understanding the genomic evolution of this taxonomically contentious lineage. The circular 16,289 bp mitogenome of *D. millardi* comprises 37 genes (13 PCGs, 22 tRNAs, and 2 rRNAs) arranged with transcriptional polarity typical of eutherian mammals [31,76], providing a molecular framework for resolving its phylogenetic placement. The genome exhibited pronounced AT bias (61.3%), aligning with strand-specific deamination patterns characteristic of murid mitogenome evolution [77]. Notably, the OL region displayed anomalous GC enrichment (58.1%), likely attributable to relaxed selective constraints on RNA primer binding during L-strand replication initiation [78]. This region’s position within the ‘WANCY’ tRNA cluster retained the ancestral eutherian ‘W-A-N-C-Y’ configuration, sharply contrasting with the derived marsupial ‘A-C-W-N-Y’ arrangement [76].

The tRNAs of *D. millardi* revealed deep evolutionary constraints through three structural hallmarks: (1) The loss of the *trnS*_1_ dihydrouridine (DHU) arm could be traced to early metazoan diversification [79] and functionally compensated by tertiary RNA structural adaptations [80]; (2) D-loop truncation of *trnK*, a synapomorphy unifying placental mammals [12], showed parallel evolution in *Microtus* and *Apodemus* lineages [81,82]; (3) most significantly, non-Watson–Crick pairing (29 G-U wobble pairs and 10 mismatches) likely stabilized tRNA architecture through low-energy hydrogen bonding [83], with potential mechanistic homology to invertebrate post-transcriptional editing processes [84]. The abovementioned mitogenomic architecture provided genomic evidence for the structural evolutionary conservation in *D. millardi*.

Crucially, we observed polyadenylation-mediated completion of truncated stop codons (T--) in *D. millardi* (Table 1), a mechanism empirically validated in *Clethrionomys glareolus* (Rodentia: Cricetidae) [85,86]. Anticodon conservation analyses further revealed that *D. millardi* retained the ancestral *trnK* (UUU) codon despite known mammalian variability [87], while its *trnS*_1_ (GCU) anticodon starkly differed from UCU variants in Siphonaptera and Arachnida [88,89]. This finding strongly suggests stringent evolutionary constraints in eutherian mitogenomes.

### 4.2. Codon Usage Bias and Evolutionary Constraints in the D. millardi Mitogenome

Analysis of RSCU across 13 PCGs revealed distinct translational preferences in *D. millardi* (RSCU = 1: neutral; RSCU > 1: preferred; RSCU < 1: avoided) [90,91]. Twenty-nine codons exhibited preference (RSCU > 1), with 13 strongly preferred codons (RSCU > 1.6) predominantly terminating in adenine (A), except UCC. Conversely, 15 codons (RSCU < 0.6) showed avoidance, primarily ending in guanine (G), excluding AGU. This bias aligns with the observed AT enrichment and GC depletion in coding regions, likely minimizing premature stop codons and preserving amino acid integrity via third-position A/T preference [92].

Purifying selection dominated mitogenome evolution (Ka/Ks < 1 for all PCGs), with divergent evolutionary rates reflecting functional constraints: *ATP8* displayed the highest rate (0.57), consistent with relaxed selective pressures in its auxiliary role [93,94], while *COX1* exhibited extreme conservation (0.03), mirroring murid-wide patterns [82]. These trends align with broader metazoan mitogenome evolution observed in Charadriiformes, Cimicomorpha, Ranidae, and other taxa [95,96,97,98].

Multivariate analyses delineated drivers of codon usage bias: (1) Functional coordination: COA revealed clustered codon preferences for *COX3*, *ND4*, and *CYTB* (Figure 7A), reflecting their coordinated roles in oxidative phosphorylation (Complexes I, III, and IV) [99]; (2) strand-specific selection: PR2 suggested *ND6*’s distinct codon usage, diverging from 12 H-strand PCGs, likely steming from L-strand-specific mutational pressures and selective optimization [100]; (3) selection dominance: ENC-plot and neutrality curve analyses confirmed natural selection as the predominant driver (80.9%) versus mutational bias (19.1%). These findings illuminate adaptive strategies shaping Murinae mitogenomes, where codon usage patterns balance structural stability, translational efficiency, and environmental adaptation—a critical framework for decoding evolutionary trajectories in the monotypic genus *Dacnomys*.

### 4.3. Phylogenetic Reconstruction and Divergence Time Estimation

In the current study, our phylogenetic analyses robustly resolved *D. millardi* as the sister lineage to *Leopoldamys* (PP = 1.00, BS = 100%), with divergence dating to the Early Pliocene (~4.8 Mya; 95% HPD: 3.65–5.47 Mya). Our results are consistent with traditional morphological classifications [1,30], obtain similar tree topologies and the stable phylogenetic placement of *Dacnomys* from different datasets [10,31].

Despite the well-established phylogenetic relationships in Murinae, the intergeneric and interspecific relationships remain contentious. A notable example is the unresolved systematic position of *Micromys*, with conflicting hypotheses emerging from different molecular datasets: (1) Sister relationship to *Rattus* (*CYTB* data) or *Tokudaisa* (*IRBP*) [101]; (2) basal positioning within the Apodemus–Rattus clade (*CYTB* analysis) [102]; (3) basal placement in tribe Rattini based on combined *CYTB* and nuclear loci [10,69]. Our mitogenome-based phylogeny and divergence dating provide robust support (PP = 1.00, BS = 98%) for *Micromys* as an early-diverging lineage (~12.9 Mya, 95% HPD: 12.24–13.57 Mya) within the Murinae, is consistent with a recent study [103] and paleontological evidence (Late Miocene origin) [104]. This contrasts with Liu et al. (2025) [25], who placed *Micromys* at the base of Rattini and identified it as sister to *Vernaya*. This topological discrepancy is likely due to methodological differences in genomic sampling: The constrained resolution for early murine radiations (e.g., *Micromys*) in Liu et al. (2025) [25] stems from limited mitogenomic representation (40 mitogenomes and 17 *CYTB* genes), whereas our comprehensive dataset (69 complete mitogenomes) provides enhanced phylogenetic signal density and branch coverage critical for resolving short internal branches [105,106]. Moreover, Pagès et al. (2015) [107] considered that the dental morphology of *Micromys* strongly differed from most other Rattini. Consequently, we support reclassifying *Micromys* from tribe Rattini to the tribe Micromyini, following the taxonomic framework of Wilson et al. (2017) [1] and the recently molecular study of Liu et al. (2025) [25], which requires further morphological validation and attention from taxonomists.

Notably, mitochondrial phylogenies inherently faced methodological constraints including incomplete lineage sorting, introgression artifacts, and hybridization signals [26,29]. While mitochondrial substitution rates (4.8 × 10^−8^ substitutions/site/year) provide superior resolution for recent radiations (faster than nuclear gene rate at 1.2 × 10^−9^) [14,108], these maternal markers alone cannot fully resolve deep divergence events like *Micromys*’s Miocene origin. We therefore advocate integrating mitogenomic and multi-locus nuclear datasets—ideally whole-genome sequences—to reconcile potential mitochondrion–nuclear gene discordance and establish a cohesive phylogenetic hypothesis.

### 4.4. Evolutionary Constraints and Anthropogenic Pressures Drive Genetic Diversity Erosion in the Threatened Murid D. millardi

As a monotypic genus, *D. millardi* exhibited distinct genetic diversity patterns, although the underlying drivers remain poorly resolved. Previous studies reported minimal cranial and body size differentiation between northern and southern Vietnamese populations of *D. millardi*, alongside low mitochondrial genetic diversity (*CYTB* divergence ≤ 0.023) [2]. However, the factors driving this homogeneity were not explicitly expatiated. To address this gap, we integrated morphological and genetic data from newly sampled populations in southwestern China (Yunnan Province), which similarly showed limited morphological variation and low genetic diversity. This pattern seems consistent with a demographic expansion following a genetic bottleneck, likely driven by post-glacial recolonization or recent habitats fragmentation [46,109]. We propose three factors underpinning *D. millardi*’s low genetic diversity, likely linked to its evolutionary history, anthropogenic pressures, and ecological specialization.

1. Habitats loss and geographic confinement synergistically drive genetic diversity erosion

Accelerating deforestation-driven habitat loss (>20,000 km^2^/year) and geographic confinement synergistically drive genetic diversity erosion in *D. millardi*—the process revealing how rapid forest degradation disproportionately threatens stenotopic species across Southeast Asia [110,111]. According to population genetic theory, genetic diversity was positively correlated with effective population size (Nₑ) and geographic range [112,113]. The species’ small, fragmented populations likely exacerbated genetic drift and inbreeding, reducing adaptive potential and amplifying extinction risks—a pattern consistent with other range-restricted murids (e.g., *Leopoldamys neilli*) [114]. Critically, low genetic diversity (π < 0.02) is a well-established hallmark of threatened mammalian species [115]. The markedly low nucleotide diversity observed in *D. millardi* (*CYTB*: *π* = 0.0135 ± 0.0023; *COX1*: *π* = 0.0101 ± 0.0025) aligns with this pattern, indicating substantial conservation concern for this taxon.

Consequently, we propose updating the conservation status of *D. millardi* and advocating for its IUCN Red List reclassification from Data Deficient (DD) to Near Threatened (NT) based on documented population declines and habitat fragmentation, while urging the integration of genetic diversity metrics into IUCN assessments to address extinction risks exacerbated by adaptive potential loss in isolated populations. Furthermore, we provide a new perspective on protecting threatened murids such as *D. millardi* by implementing protection of core habitats to prevent population collapses driven by deforestation.

2. Shallow evolutionary history

Divergence time estimation (by BEAST) revealed that *Dacnomys* split from its sister genus *Leopoldamys* in the early Pliocene (~4.8 Mya; 95% HPD: 3.65–5.47 Mya). This relatively shallow evolutionary history may have limited time for mutation accumulation compared to polytypic genera lineages [109], which might have a longer evolutionary history, accumulate more genetic variations, and exhibit higher genetic diversity [116,117]. Short evolutionary histories often constrain genetic variation in monotypic genera due to insufficient time for lineage-specific substitution [118], particularly under stabilizing selection in special environments.

3. Ecological specialization and adaptive stability

As a threatened lineage endemic to the Indo-Malayan biodiversity hotspot—a proposed center of Murinae diversification [69]—*D. millardi* has likely undergone long-term adaptation to stable montane rainforest niches. Specialization to narrow ecological optima could reduce selective pressures for novel genetic variation, favoring purifying selection to preserve adaptive traits while eliminating deleterious alleles [119,120]. This process, combined with environmental stability in undisturbed habitats where *D. millardi* has only been discovered, might explain the observed genetic homogeneity, as the species’ genomic architecture reflects optimization for its ancestral niche rather than adaptive flexibility.

## 5. Conclusions

Our study combines morphological characterization with the complete mitogenome to validate the phylogenetic placement of *Dacnomys* and reconstruct the phylogenetic framework of the subfamily Murinae. By coupling the complete mitogenome, genetic diversity, and codon usage bias analysis, we reveal the following. (1) Phylogenetic reassessment: *Dacnomys* forms a well-supported sister clade to *Leopoldamys* (PP = 1.0, BS = 100%), while the basal positioning of *Micromys* within Murinae necessitates its elevation to the tribal rank Micromyini, which requires further morphological validation and attention from taxonomists. These findings refine the subfamily’s classification and highlight the evolutionary uniqueness of the understudied monotypic genus *Dacnomys*. (2) Evolutionary constraints: strong purifying selection (Ka/Ks < 1) dominates mitogenome evolution, as evidenced by codon usage patterns. This intrinsic constraint, coupled with the lineage’s recent Pliocene divergence (~4.8 Mya), limits mutational accumulation, resulting in reduced nucleotide diversity. (3) Synergistic risks: habitat fragmentation and population decline—driven by Southeast Asia’s unparalleled deforestation rates (>20,000 km^2^/year since 2000)—amplify genetic drift in *D. millardi*’s small, isolated populations. The interaction of these extrinsic pressures with evolutionary constraints creates a dual erosion trap, depleting adaptive potential and escalating extinction vulnerability. These findings necessitate urgent conservation prioritization for threatened murids by implementing protection of core habitats to prevent deforestation-driven population collapses and advocating IUCN Red List reclassification of *D. millardi* from Data Deficient (DD) to Near Threatened (NT) with integration of genetic diversity metrics into IUCN Red List assessments.

## Figures and Tables

**Table 1:** Structural characteristics and annotation of the *Dacnomys millardi* mitogenome.

Gene	Strand	Position (bp)	Size (bp)	Start Codon	Stop Codon	Anticodon	Intergenic Nucleotides
*trnF*	H	1–69	69			GAA	0
*rrnS*	H	70–1023	954				0
*trnV*	H	1024–1092	69			UAC	0
*rrnL*	H	1093–2668	1576				0
*trnL*_1_	H	2667–2741	75			UAG	−2
*ND1*	H	2739–3698	960	ATA	TAG		−3
*trnI*	H	3697–3765	69			GAU	−2
*trnQ*	L	3763–3833	71			UUG	−3
*trnM*	H	3837–3905	69			CAU	3
*ND2*	H	3906–4940	1035	ATT	AGA		0
*trnW*	H	4942–5007	66			UCA	1
*trnA*	L	5009–5077	69			UGC	1
*trnN*	L	5079–5149	71			GUU	1
OL	L	5150–5180	31				0
*trnC*	L	5181–5247	67			GCA	0
*trnY*	L	5248–5314	67			GUA	0
*COX1*	H	5316–6860	1545	ATG	TAA		1
*trnS*_2_	L	6858–6926	69			UGA	−3
*trnD*	H	6930–6998	69			GUC	3
*COX2*	H	7000–7683	684	ATG	TAA		1
*trnK*	H	7687–7750	64			UUU	3
*ATP8*	H	7752–7955	204	ATG	TAA		1
*ATP6*	H	7913–8593	681	ATG	TAA		−43
*COX3*	H	8593–9376	784	ATG	T--		−1
*trnG*	H	9377–9444	68			UCC	0
*ND3*	H	9445–9792	348	ATC	TAA		0
*trnR*	H	9794–9862	69			UCG	1
*ND4L*	H	9865–10,161	297	ATG	TAA		2
*ND4*	H	10,155–11,532	1378	ATG	T--		−7
*trnH*	H	11,533–11,600	68			GUG	0
*trnS*_1_	H	11,601–11,659	59			GCU	0
*trnL*_2_	H	11,659–11,729	71			UAA	−1
*ND5*	H	11,736–13,559	1824	ATA	TAA		6
*ND6*	L	13,537–14,055	519	ATG	TAA		−23
*trnE*	L	14,056–14,124	69			UUC	0
*CYTB*	H	14,130–15,273	1144	ATG	T--		5
*trnT*	H	15,274–15,340	67			UGU	0
*trnP*	L	15,341–15,408	68			UGG	0
D-loop	H	15,409–16,289	881				0

**Table 2:** Nucleotide composition and AT-GC skew of the *Dacnomys millardi* mitogenome.

*Dacnomys millardi*	Size (bp)	A%	T%	G%	C%	A + T%	G + C%	AT Skew	GC Skew
Mitogenome	16,289	33.9	28.1	12.1	25.9	62.0	38.0	0.094	−0.363
PCGs	11,400	31.6	29.7	11.7	27.0	61.3	38.7	0.031	−0.396
rRNAs	2530	38.2	24.9	16.6	20.3	63.1	36.9	0.211	−0.099
tRNAs	1503	34.4	31.3	17.8	16.6	65.7	34.4	0.048	0.035
D-loop	881	33.6	29.3	11.6	25.5	62.9	37.1	0.068	−0.375
OL	31	25.8	16.1	29.0	29.1	41.9	58.1	0.232	−0.002

**Table 3:** Codon frequency and relative synonymous codon usage (RSCU) for 13 protein-coding genes (PCGs) in the *Dacnomys millardi* mitogenome.

Codon	No.	RSCU	Codon	No.	RSCU	Codon	No.	RSCU	Codon	No.	RSCU
UUU(F)	107	0.92	GUG(V)	6	0.15	CAU(H)	37	0.71	UGG(W)	4	0.08
**UUC(F)**	**126**	**1.08**	CCU(P)	44	0.98	**CAC(H)**	**67**	**1.29**	CGU(R)	11	0.69
**UUA(L)**	**122**	**1.23**	**CCC(P)**	**60**	**1.21**	**CAA(Q)**	**80**	**1.95**	CGC(R)	11	0.69
UUG(L)	11	0.11	**CCA(P)**	**92**	**1.86**	CAG(Q)	2	0.05	**CGA(R)**	**37**	**2.31**
CUU(L)	86	0.87	CCG(P)	2	0.04	AAU(N)	60	0.73	CGG(R)	5	0.31
**CUC(L)**	**103**	**1.04**	ACU(T)	60	0.80	**AAC(N)**	**104**	**1.27**	GGU(G)	39	0.69
**CUA(L)**	**263**	**2.65**	**ACC(T)**	**85**	**1.12**	**AAA(K)**	**96**	**1.94**	**GGC(G)**	**58**	**1.11**
CUG(L)	10	0.10	**ACA(T)**	**155**	**2.04**	AAG(K)	3	0.06	**GGA(G)**	**101**	**1.93**
**AUU(I)**	**201**	**1.02**	ACG(T)	3	0.04	GAU(D)	26	0.72	GGG(G)	14	0.27
AUC(I)	195	0.98	**GCU(A)**	**58**	**1.01**	**GAC(D)**	**46**	**1.28**	UCU(S)	35	0.69
**AUA(M)**	**193**	**1.75**	**GCC(A)**	**90**	**1.57**	**GAA(E)**	**84**	**1.75**	**UCC(S)**	**92**	**1.80**
AUG(M)	28	0.25	**GCA(A)**	**78**	**1.36**	GAG(E)	12	0.25	**UCA(S)**	**122**	**2.39**
**GUU(V)**	**43**	**1.09**	GCG(A)	4	0.07	UGU(C)	12	0.83	UCG(S)	3	0.06
GUC(V)	31	0.78	UAU(Y)	62	0.98	**UGC(C)**	**17**	**1.17**	AGU(S)	17	0.33
**GUA(V)**	**78**	**1.97**	**UAC(Y)**	**64**	**1.02**	**UGA(W)**	**100**	**1.92**	AGC(S)	37	0.73

Notes: Termination codons were excluded from analysis. Preferred codons (RSCU > 1.0) are in bold, over-represented codons (RSCU > 1.6) are highlighted in red, and under-represented codons (RSCU < 0.6) are presented in blue.

## Data Availability

The experimental data used to support the findings of this study are available from GenBank of NCBI: https://www.ncbi.nlm.nih.gov/ (accessed on 5 June 2025) for the complete mitogenome of *D. millardi* (accession number: PQ359525). The two specimens of *D. millardi* are also available from the Institute of Pathogens and Vectors, Dali University, China (voucher IDs: E420001, E420002).

## Supplementary Materials

The following supporting information can be downloaded at: https://www.mdpi.com/article/10.3390/biology14080948/s1, Table S1: Geographic coordinates of sampling sites from this study and Abramov et al. (2017) [2]; Table S2: Detailed information of the 18 *Dacnomys millardi* DNA sequences (nine *CYTB* and nine *COX1*) from Abramov et al. (2017) [2] used for genetic diversity analysis, including GenBank accession numbers and geographic distribution; Table S3: Mitochondrial genomes used in phylogenetic analyses of Muridae, with specimen collection localities and GenBank accession numbers; Table S4: The test results of substitution saturation based on partitioned mitochondrial PCGRNA alignment (13,735 bp); Table S5: Fossil constraints and node calibrations for divergence dating of Muridae, including fossil taxa, phylogenetic placement, and geological age (Mya); Table S6: External and craniometric measurements (mm, g) of adult *Dacnomys millardi* specimens from Weixi Lisu Autonomous County, Yunnan Province, China (27.18° N, 99.28° E; voucher IDs: E420001, E420002). Specimen E420002 exhibited bilateral broken zygomatic arches, compromising the accuracy of key metrics like orbital length and zygomatic breadth. Damaged measurements flagged with ‘*’. The intact specimen (E420001) is retained in the study; Table S7: Intraspecific genetic divergence of *Dacnomys millardi* across Southwestern China, northern and southern Vietnam inferred from mitochondrial *CYTB* sequences; Table S8: Genetic diversity metrics of mitochondrial *CYTB* and *COX1* genes in *Dacnomys millardi* populations from China (Yunnan Province) and Vietnam.
