# Supplementary material for: Phylogenetic Reassessment of Murinae Inferred from the Mitogenome of the Monotypic Genus Dacnomys Endemic to Southeast Asia: New Insights into Genetic Diversity Erosion
Source: Biology (Basel). 2025 Jul 28;14(8):948. doi: 10.3390/biology14080948 (PMC12383712; doi:10.3390/biology14080948)
Supplement: Supplementary file 1 [file biology-14-00948-s001.zip › Supplementary.pdf]

SUPPLEMENTARY INFORMATION

Table S1. Geographic coordinates of sampling sites from this study and Abramov et al. (2017).

Coordinate	Categories	Latitude	Longitude	Region
1	This study (morphology and mitogenome)	27.183333	99.283333	Southwestern China
2	Published genetic samples (*CYTB* and *COX1*)	14.722222	108.316111	Southern Vietnam
3	Published genetic samples (*CYTB* and *COX1*)	22.365	102.2375	Northern Vietnam
4	Published genetic samples (*CYTB* and *COX1*)	22.411917	103.740056	Northern Vietnam
5	Published genetic samples (*CYTB* and *COX1*)	21.139583	104.768833	Northern Vietnam
6	Published genetic samples (CYTB and *COX1*)	21.136389	104.936389	Northern Vietnam
7	Published genetic samples (*CYTB* and *COX1*)	21.45	105.64	Northern Vietnam
8	Museum vouchers (only morphology)	27.416667	87.1	Eastern Nepal
9	Museum vouchers (only morphology)	26.966667	88.366667	India
10	Museum vouchers (only morphology)	26.716667	88.4	India
11	Museum vouchers (only morphology)	25.466667	91.766667	India
12	Museum vouchers (only morphology)	25.3	94.05	India
13	Museum vouchers (only morphology)	28.783333	95.916667	India
14	Museum vouchers (only morphology)	22.023333	102.1	Northern Laos
15	Museum vouchers (only morphology)	22.35	103.866667	Northern Vietnam
16	Museum vouchers (only morphology)	21.973889	104.041111	Northern Vietnam
17	Museum vouchers (only morphology)	21.337	104.60825	Northern Vietnam
18	Museum vouchers (only morphology)	21.343056	104.748611	Northern Vietnam
19	Museum vouchers (only morphology)	21.05	104.716667	Northern Vietnam
20	Museum vouchers (only morphology)	22.883333	105.733333	Northern Vietnam

Table S2. Detailed information of the 18 *Dacnomys millardi* DNA sequences (nine *CYTB* and nine *COX1*) from Abramov et al. (2017) used for genetic diversity analysis, including GenBank accession numbers and geographic distribution.

<table>
<tr><th>Dacnomys millardi</th><th>CYTB</th><th>COX1</th><th>Distribution</th></tr>
<tr><td>1</td><td>JQ755896</td><td>JQ755850</td><td rowspan="7">Northern Vietnam</td></tr>
<tr><td>2</td><td>JQ755897</td><td>JQ755851</td></tr>
<tr><td>3</td><td>KC878029</td><td>KU958013</td></tr>
<tr><td>4</td><td>KX977415</td><td>KX977409</td></tr>
<tr><td>5</td><td>KX977416</td><td>KX977410</td></tr>
<tr><td>6</td><td>KX977417</td><td>KX977411</td></tr>
<tr><td>7</td><td>KX977418</td><td>KX977414</td></tr>
<tr><td>8</td><td>KX977419</td><td>KX977412</td><td rowspan="2">Southern Vietnam</td></tr>
<tr><td>9</td><td>KX977420</td><td>KX977413</td></tr>
</table>

Table S3. Mitochondrial genomes used in phylogenetic analyses of Muridae, with specimen collection localities and GenBank accession numbers.

Genus	Species and subspecies	Accession No.	country	locality
Apodemus	*Apodemus agrarius*	NC_016428.1	South Korea	Mt. Jiri, Jeollanam-do
Apodemus	*Apodemus chejuensis*	NC_016662.1	South Korea	/
Apodemus	*Apodemus chevrieri*	NC_017599.1	China	Sichuan Province
Apodemus	*Apodemus peninsulae*	NC_016060.1	South Korea	/
Apodemus	*Apodemus draco*	NC_019584.1	China	Gong ga Mountain
Apodemus	*Apodemus latronum*	NC_019585.1	China	Gong ga Mountain
Apodemus	*Apodemus flavicollis*	MN122902.1	Denmark	/
Apodemus	*Apodemus sylvaticus*	NC_049122.1	France	/
Rattus	*Rattus baluensis*	NC_035621.1	Malaysia	Sabah, summit trail Mt Tambuyucon
Rattus	*Rattus tiomanicus*	NC_029888.1	Malaysia	/
Rattus	*Rattus rattus*	NC_012374.1	New Zealand	/
Rattus	*Rattus tanezumi*	NC_011638.1	Japan	Amami Is
Rattus	*Rattus hoogerwerfi*	NC_049040.1	Indonesia	/
Rattus	*Rattus andamanensis*	NC_046686.1	China	Guangdong Province
Rattus	*Rattus exulans*	NC_012389.1	Thailand	/
Rattus	*Rattus nitidus*	NC_040919.1	China	Tibet
Rattus	*Rattus norvegicus*	NC_001665.2	/	Wistar: type A
Rattus	*Rattus sordidus*	NC_014871.1	Australia	Northern Territory
Rattus	*Rattus villosissimus*	NC_014864.1	Australia	South Australia
Rattus	*Rattus lutreolus*	NC_014858.1	Australia	Tasmania
Rattus	*Rattus tunneyi*	NC_014861.1	Australia	Northern Territory
Rattus	*Rattus fuscipes*	NC_014867.1	Australia	Western Australia
Rattus	*Rattus leucopus*	NC_014855.1	Papua New Guinea	Central Highland
Rattus	*Rattus niobe*	NC_023347.1	Papua New Guinea	Sol River, West Sepik Province
Rattus	*Rattus praetor*	NC_012461.1	Papua New Guinea	Wigote
Bandicota	*Bandicota bengalensis*	NC_057104.1	China	Deihong, Yunnan Province
Bandicota	*Bandicota indica*	KT029807.1	China	Guangxi Province
Berylmys	*Berylmys berdmorei*	NC_036730.1	Thailand	/
Niviventer	*Niviventer confucianus*	NC_023960.1	China	Shangdong Province
Niviventer	*Niviventer sacer*	MZ935252.1	China	Ai Mountain, Shangdong Province
Niviventer	*Niviventer lotipes*	NC_065402.1	China	Hainan Province
Niviventer	*Niviventer andersoni*	NC_060500.1	China	Lu feng, Yunnan province
Niviventer	*Niviventer excelsior*	NC_019617.1	China	Sichuan Province
Niviventer	*Niviventer cremoriventer*	NC_035822.1	Malaysia	/
Niviventer	*Niviventer fulvescens*	NC_028715.1	China	Sichuan Province
Leopoldamys	*Leopoldamys edwardsi*	NC_025670.1	China	Sichuan Province
Leopoldamys	*Leopoldamys sabanus*	NC_035819.1	Malaysia	/

Maxomys	*Maxomys surifer*	NC_036732.1	Thailand	/
Maxomys	*Maxomys whiteheadi*	NC_049119.1	Thailand	/
Maxomys	*Maxomys ochraceiventer*	NC_056988.1	Malaysia	Sabah
Mus	*Mus musculus domesticus*	NC_006914.1	/	Strain: AKR/J
Mus	*Mus musculus helgolandicus*	KP877610.1	Germany	Heligoland Island
Mus	*Mus musculus molossinus*	NC_006915.1	Japan	/
Mus	*Mus musculus musculus*	NC_010339.1	Czech Republic	Kunratice
Mus	*Mus macedonicus*	NC_072557.1	Tanzania	Mkundi
Mus	*Mus spretus*	NC_025952.1	Europe	/
Mus	*Mus fragilicauda*	NC_025287.1	South-East Asia	/
Mus	*Mus terricolor*	NC_010650.1	American	/
Mus	*Mus cookii*	NC_025270.1	South-East Asia	/
Mus	*Mus famulus*	NC_030342.1	India	Nilgiri Mountains
Mus	*Mus cervicolor*	NC_025269.1	South-East Asia	/
Mus	*Mus caroli*	NC_025268.1	South-East Asia	/
Mus	*Mus minutoides*	NC_072557.1	Tanzania	Mkundi
Mus	*Mus pahari*	NC_036680.1	Australia	/
Tokudaia	*Tokudaia osimensis*	LC642727.1	Japan	Kagoshima, Amami-oshima island
Arvicanthis	*Arvicanthis rufinus*	NC_053802.1	Czech Republic	/
Arvicanthis	*Arvicanthis somalicus*	NC_053801.1	Czech Republic	/
Chiropodomys	*Chiropodomys gliroides*	NC_049121.1	African	/
Micromys	*Micromys erythrotis*	NC_060316.1	China	Guizhou Province
Micromys	*Micromys minutus*	NC_027932.1	China	Sichuan Province
Hapalomys	*Hapalomys delacouri*	MZ159976.1	African	/
Meriones	*Meriones meridianus*	NC_027684.1	China	/
Meriones	*Meriones unguiculatus*	KF425526.1	China	/
Meriones	*Meriones libycus*	NC_027683.1	China	/
Brachiones	*Brachiones przewalskii*	KT834972.1	China	/
Meriones	*Meriones tamariscinus*	NC_034314.1	China	Khorgos, Xingjiang Province
Rhombomys	*Rhombomys opimus*	MK359635.1	China	/
Ratufa	*Ratufa bicolor*	NC_023780.1	China	Hainan Province
Pteromys	*Pteromys volans*	NC_019612.1	South Korea	/

Notes: List of 68 mitochondrial genomes (including four *Mus musculus* subspecies) from Muridae, with collection country, locality, and GenBank accession numbers. Specimens without locality data are marked '/'. Data were retrieved from GenBank on [25/11/2024], excluding sequences with missing genes or ambiguous annotations. The final dataset spans 16 genera, 8 tribes, and 2 subfamilies, rooted with *Ratufa bicolor* and *Pteromys volans* outgroups (NCBI accessions: NC_023780.1, NC_019612.1)

Table S4. The test results of substitution saturation based on partitioned mitochondrial PCGRNA alignment (13,735 bp).

Codon position	NumOTU	Iss	Iss.cSym	P	Iss.cAsym	P
All sites	4	0.674	0.859	0.0000	0.847	0.0000
	8	0.693	0.844	0.0000	0.761	0.0000
	16	0.707	0.853	0.0000	0.675	0.0000
	32	0.736	0.819	0.0000	0.573	0.0000
1&2nd	4	0.160	0.856	0.0000	0.846	0.0000
	8	0.157	0.845	0.0000	0.764	0.0000
	16	0.172	0.846	0.0000	0.678	0.0000
	32	0.187	0.816	0.0000	0.571	0.0000
3rd	4	0.493	0.850	0.0000	0.841	0.0000
	8	0.487	0.845	0.0000	0.765	0.0000
	16	0.492	0.830	0.0000	0.677	0.0000
	32	0.514	0.810	0.0000	0.562	0.0000

Note: Analysis performed on all sites for 1&2nd and 3rd codon position separately. Iss, index of substitution saturation; IssSym is Iss.c assuming a symmetrical topology, IssAsym is Iss.c assuming an asymmetrical topology; NumOTU, number of operation taxonomic units.

Table S5. Fossil constraints and node calibrations for divergence dating of Muridae, including fossil taxa, phylogenetic placement, and geological age (Mya).

	Fossil	Placement	Age (Mya)	Reference
1	†*Antemus chinjiensis*	crown Murinae	13.8	Aghová et al. (2018)
2	†*Parapodemus lugdunensis*	stem Apodemini	9.6	
3	†*Mus sp.*	stem MRCA *Mus*	8.0	
4	†*Arvicanthis sp.*	stem MRCA *Arvicanthis*	6.1	

Table S6. External and craniometric measurements (mm, g) of adult *Dacnomys millardi* specimens from Weixi Lisu Autonomous County, Yunnan Province, China (27.18° N, 99.28° E; voucher IDs: E420001, E420002). Specimen E420002 exhibited bilateral broken zygomatic arches, compromising the accuracy of key metrics like orbital length and zygomatic breadth. Damaged measurements flagged with '*'. The intact specimen (E420001) is retained in the study.

Characters	*Dacnomys millardi* (n=2)		
	E420001	E420002	Mean
Greatest length of skull	54.91	54.50	54.71
Condylobasal length	51.63	52.52	52.08
Height of braincase	15.56	15.42	15.49
Breadth of braincase	19.83	19.76	19.80
Occipitonasal length	56.65	56.01	56.33

Length of nasals	19.63	19.74	19.69
Breadth of nasals	6.30	6.98	6.64
Palate length	30.45	31.03	30.74
Post palatal length	23.11	23.76	23.44
Interorbital breadth	7.75	7.91	7.83
Orbital length	20.12	21.34*	20.73*
Zygomatic breadth	27.27	28.63*	27.95*
Length of incisor	9.98	8.74	9.36
Breadth of incisor	3.97	4.42	4.20
Length of incisive foramina	10.21	11.51	10.86
Breadth of incisive foramina	4.25	4.35	4.30
Length of upper tooth row	31.19	31.65	31.42
Length of upper molar row	11.29	12.04	11.67
Length of auditory bulla	7.63	7.29	7.46
Breadth of auditory bulla	4.47	4.10	4.29
Height of mandible	16.44	15.34	15.89
Length from the condyle	30.26	31.05	30.66
Length of the tooth row	19.66	19.87	19.77
Length of the molar row	11.30	11.04	11.17
Head-body length	270.00	260.00	265.00
Tail length	305.00	295.00	300.00
Ear length	24.00	23.00	23.50
Hind foot length	50.00	50.00	50.00
Body weight	475.00	515.00	495.00

Note: Craniometric measurements followed Yang (2005; https://doi.org/10.13859/j.cjz.2005.03.011) for protocols. The specimens (voucher IDs: E420001, E420002) were stored in the Institute of Pathogens and Vectors, Dali University (Dali, China).

Table S7. Intraspecific genetic divergence of *Dacnomys millardi* across Southwestern China, northern and southern Vietnam inferred from mitochondrial *CYTB* sequences.

Dacnomys millardi	Distribution		1	2	3	4	5	6	7	8	9
JQ755896	Northern Vietnam	1									
JQ755897		2	0.001								
KC878029		3	0.017	0.015							
KX977415		4	0.017	0.015	0.008						
KX977416		5	0.008	0.006	0.007	0.007					
KX977417		6	0.003	0.001	0.011	0.011	0.004				
KX977418		7	0.003	0.001	0.011	0.011	0.004	0.000			
KX977419	Southern Vietnam	8	0.023	0.022	0.014	0.017	0.014	0.018	0.018		
KX977420		9	0.022	0.021	0.013	0.016	0.013	0.017	0.017	0.002	
PQ359525	Southwestern China	10	0.021	0.019	0.016	0.016	0.013	0.016	0.016	0.020	0.019

Note: Pairwise genetic distances calculated under the Tamura 3-parameter model (Tamura, 1992) with gamma correction (α = 0.5) in MEGA v11.0.13. Samples include one sequence from Southwestern China (Yunnan: PQ359525), two from Southern Vietnam (Kon Tum: KX977419–KX977420), and seven from Northern Vietnam (Son La: JQ755896–JQ755897; Vinh Phuc: KC878029; Dien Bien: KX977415–KX977418).

Table S8. Genetic diversity metrics of mitochondrial *CYTB* and *COX1* genes in *Dacnomys millardi* populations from China (Yunnan Province) and Vietnam.

Gene	Number of sequences	Number of haplotypes	Number of sites, excluding sites with gaps / missing data	Variable (polymorphic) sites /Total number of mutations	Singleton variable sites	Parsimony informative sites	Number of polymorphic (segregating) sites, S	Mean number of pairwise differences, K	Haplotype (gene) diversity, *Hd*	St. dev. of *Hd*	Nucleotide diversity, π	St. dev. of π	Tajima's D
CYTB	10	9	1144/1011	42/42	9	33	42	13.62222	0.978	0.054	0.01347	0.00227	-0.40094
COX1	10	6	1556/627	19/20	6	13	19	6.33333	0.844	0.103	0.01010	0.00246	-0.48964

Note: Summary statistics for intraspecific DNA polymorphism in 10 mitochondrial sequences per gene (*CYTB*: 1144 bp; *COX1*: 1556 bp) of *Dacnomys millardi*. Analyses included haplotype diversity (*Hd*), nucleotide diversity (π), and Tajima’s D, calculated in DnaSP v6.12.03 (Rozas et al., 2017). Samples comprise one sequence from Yunnan (GenBank accession: PQ359525), four from Southern Vietnam (Kon Tum: *CYTB* KX977419–977420; *COX1* KX977412–KX977413), and fourteen from Northern Vietnam (Son La: *CYTB* JQ755896–JQ755897, *COX1* JQ755850–JQ755851; Vinh Phuc: *CYTB* KC878029; Dien Bien: *CYTB* KX977415–KX977418, *COX1* KU958013; Lao Cai: *COX1* KX977409, KX977414; Phu Tho: *COX1* KX977410–KX977411). Tajima’s D values (*CYTB*: D = -0.40094; *COX1*: D = -0.48964) were non-significant ($P > 0.05$).
